# Comparison of Cardiorespiratory Fitness of Chinese Tibetan Adolescents with Their Han Counterparts: A Cross-Sectional Retrospective Study

**DOI:** 10.3390/ijerph192416526

**Published:** 2022-12-09

**Authors:** Li Zhang, Ruming Zhang, Feng Zhang, Xiaojian Yin, Yuan Liu, Yaru Guo, Pengwei Sun

**Affiliations:** 1 Department of Physical Education, China University of Mining and Technology, Beijing 100083, China; 2 Key Laboratory of Adolescent Health Assessment and Exercise Intervention of Ministry of Education, East China Normal University, Shanghai 200241, China; 3 College of Physical Education and Health, East China Normal University, Shanghai 200241, China; 4 College of Economics and Management, Shanghai Institute of Technology, Shanghai 201418, China

**Keywords:** cardiorespiratory fitness, Tibetan, Han, VO_2max_, adolescents

## Abstract

Cardiorespiratory fitness (CRF) is a core element of healthy physical fitness. Foreign attention to CRF in adolescents at different altitudes is high, while less research has been conducted on Chinese adolescents. In order to compare the CRF of Chinese Tibetan adolescents with their Han counterparts born and raised at high altitude and Chinese Han adolescents at sea level. A total of 2748 participants, including Chinese Tibetan adolescents, Chinese Han adolescents born and raised at high altitudes, and Chinese Han adolescents at sea level aged 12–18 years old, were obtained using convenience sampling and random cluster sampling. The method of the 20 m shuttle run test (20 m SRT) test was used to derive VO_2max_ by equation. One-way ANOVA and LSD methods were conducted, and effect sizes were calculated to compare the CRF of the three types of adolescents. Regression analysis was used to analyze the relationship between altitude and VO_2max_. The VO_2max_ scores of Chinese Tibetan adolescents and Chinese Han adolescents at sea level were higher than Chinese Han adolescents born and raised at high altitudes. For both boys and girls, the VO_2max_ scores of Chinese Tibetan adolescents exceeded Chinese Han adolescents at sea level after the age of 16 years old. Regression analysis showed that altitude was inversely associated with VO_2max_. The pace of lung growth may distinguish Chinese Tibetan adolescents from Chinese Han adolescents born and raised at high altitudes. The results of the study suggest that we should focus on the changes in CRF in adolescents at different altitudes and should adopt different CRF interventions for adolescents at different altitudes.

## 1. Introduction

There are about 140 million people around the world living at 2500 m above sea level [1]. Living at a high altitude is a great challenge to human survival and reproduction. The extreme conditions include hypobaric hypoxia, low temperatures and relative humidity, high cosmic radiation, and so on [2,3,4]. Located in China’s Qinghai-Tibet Plateau, which is known as the ‘roof of the world,’ Chinese Tibetans have lived at 3400 m above sea level for about 30,000 years and physiologically adapted to the hypoxic environment [5,6,7]. Many studies have been published concentrated on the growth [8,9] and the poor nutritional status [10,11] of Tibetan children and adolescents. Anyway, indicators related to lung function, such as forced vital capacity, and cardiorespiratory fitness (CRF), were also concerned since their sensitivity to hypoxia [12,13,14]. For example, Chen et al. [14] concluded that exposure to high altitude from birth to adolescence resulted in efficient O2 transport and greater aerobic exercise performance that may reflect a successful adaptation to life at high altitude.

It should be noted that several studies focused on the comparison between Tibetans and Han nationality who was born and raised at high altitude [15,16,17]. For example, Weitz et al. [17] compared forced vital capacity (FVC) and Forced Expiratory Volume at 1 Second (FEV1) of Chinese Tibetans in Qinghai province with those Han who were born and raised at high altitudes, found out that the FVC and FEV1 values of Tibetans are generally greater than those of Han born and raised at high altitude, and the differences were significantly larger among older adolescents and adults. The study also confirmed that Tibetan newborns in Lhasa had higher arterial oxygen saturation at birth and during the first four months of life than Han newborns [18]. However, forced vital capacity can reflect the healthy fitness of adolescents to some extent. However, CRF, as the core element of healthy physical fitness, also better reflects the level of healthy physical fitness of adolescents. Unfortunately, there are few studies on adolescents in high-altitude areas in China.

CRF involves an individual’s ability to deliver oxygen from the atmosphere to the mitochondria to perform physical work [19] and has been known as an important predictor of cardiovascular disease risk, cancer, executive functions, academic achievement, and mental health in children and adolescents [20,21,22,23]. Therefore, it is an important determinant of one’s ability to thrive at a high altitude owing to the hypoxia condition. Fan et al. compared the CFR of Tibetan children and adolescents to their Han Chinese counterparts in Shanghai [24], while Han born and raised at high altitude was not involved. The present study aimed to compare the CRF of Chinese Tibetan adolescents with their Han counterparts born and raised at high altitudes and Chinese Han adolescents at sea level. Our study hypothesized that there were differences in CRF among adolescents at different altitudes, with Chinese Han adolescents at sea level having the highest CRF.

## 2. Materials and Methods

### 2.1. Study Design and Population

The research was carried out from September to December 2019. Participants involved in the present study should be born and live in the local area for at least two years without physical or mental diseases. Our study was conducted using a cross-sectional retrospective study method. A total of 2748 participants (1447 boys and 1301 girls) aged 12–18 years old were obtained. (1) Chinese Tibetan adolescents: according to Tibet’s geographical environment, three cities (Lhasa, Nagqu, and Changdu) 3600 m above sea level were selected, and six schools were selected from these three cities. The selection of schools in each city is based on the distribution of urban and rural areas and the distribution of geographical locations. In each school, classes were randomly selected as the minimum sampling unit. At last, 864 valid data were collected (439 boys and 425 girls). (2) Chinese Han adolescents born and raised at high altitude: Chinese Han adolescents born and raised at high altitude were selected from two schools in Lhasa for investigation. The method of selecting classes was the same as that of Chinese Tibetans. At last, 898 valid data were collected (452 boys and 446 girls). (3) Chinese Han adolescents at sea level: stratified cluster random sampling method was adopted to select 4780 adolescents aged 12–18 years old from seven administrative regions of China (Shanghai and Jiangxi province in East China, Guangdong, and Guangxi province in South China, Hunan province and Hubei province in Central China’s, Shanxi Province in North China, Heilongjiang Province in Northeast China, Xinjiang Uygur Autonomous Region in Northwest China, Sichuan and Yunnan provinces in southwest China). All the involved provinces are in plain areas of China. Data for Chinese Han adolescents at sea level (986 adolescents, 556 boys and 430 girls) were randomly selected from this study.

This study was approved by the Human Ethics Committee of East China Normal University (Approval Number: HR0782020). Before the investigation, written informed consent was obtained from students and their parents. Participants’ names were encrypted by number to strictly protect their privacy.

### 2.2. Measurements

This research was conducted by qualified personnel who have been uniformly trained before in advance. The testers were divided into three groups and entered the school for investigation. Anthropometric information such as age, ethnicity, and residence time at the local region was collected. CRF was assessed by 20 m SRT (20 m shuttle run test), which has good test validity and retest reliability(*r* = 0.78, 0.72–0.85) (*r* = 0.72, *p* < 0.001) [25,26]. The 20 m SRT is currently the most popular field assessment method for CRF in children and adolescents worldwide [27,28]. The specific test was conducted at the school’s athletic field. The test is a round trip run between two lines 20 m apart. The test was conducted on music. The test was started when the subject heard the “ding dong” tone and ran to the opposite end of the line, and then ran to the opposite end of the line when the “ding dong” tone was heard again, and so on. The test was completed when the participant did not reach the opposite end line after two consecutive tones, and the number of laps was recorded with the last one to obtain the final grade. Based on this result, the maximum oxygen uptake was calculated through an equation. The detailed measuring method was provided by our previous publishers [29,30]. Maximum oxygen uptake (VO_2max_) was evaluated from laps of 20 m SRT by Leger equation(r = 0.79) [31]:

VO_2max_ = 31.025 + 3238 × S − 3248 × Age + 0.1536 × S × Age,

S: the running speed at the last completed stage (km h^−1^); S = 8 + 0.5 × the highest level.

Age: age on the last birthday.

### 2.3. Statistical Analyses

Age was calculated based on their last birthday. For example, 12 years old means 12.0–12.9 years old. The 20 m SRT laps were expressed as integers (Table 1). The 20 m SRT was expressed as mean and standard deviation (SD) by gender and age. One-way ANOVA and LSD methods were conducted to compare CRF between Chinese Tibetan adolescents, Chinese Han adolescents born and raised at high altitudes, and Chinese Han adolescents at sea level. The results also include eta square. Taking the VO_2max_ means of Chinese Tibetan adolescents as references, the effect size was obtained, and the effect size for the difference between different groups was calculated by Cohen’s d (small effect = 0.2; medium effect = 0.5; large effect = 0.8) [32]. Taking Chinese Han adolescents at sea level as a reference, Chinese Han adolescents born and raised at high altitudes and Chinese Tibetan adolescents were set as dummy variables of 0 and 1, with VO_2max_ as the dependent variable and altitude as the independent variable for linear regression analysis. The difference is statistically significant with *p* < 0.05. Data analysis was processed by IBM SPSS25.0 (version 25.0; IBM Inc., Armonk, NY, USA) and GraphPad Prism 8.0.2 (GraphPad Software, Inc., San Diego, CA, USA).

## 3. Results

Table 1 shows the 20 m SRT laps for the three types of adolescents. It can be concluded that, for boys, the Chinese Han adolescents born and raised at high altitudes performed worst among the three groups. The 20 m SRT laps of Chinese Tibetan adolescents were lower than Chinese Han adolescents at sea level before 16 years old but higher in 17–18 years old groups. Similar patterns were found in girls except in 12 years old group in which Chinese Han adolescents born and raised at high altitudes performed best. Chinese Tibetan adolescents catch up with Chinese Han adolescents at sea level at 16–17 years old groups and exceed them at 18 years old by three laps.

Table 2 shows the VO_2max_ scores of boys aged 12–18 for the three types of adolescents in China. One-way ANOVA shows that there are significant differences in the VO_2max_ scores for the three types of adolescents in every age group from 12 to 18 years old (*p* < 0.01). For each type of adolescent, there are significant differences in VO_2max_ scores among different ages (*p* < 0.01). In general, Chinese Han adolescents born and raised at high altitudes performed worst, with the biggest difference at 18 years old, which is 6.15 kg^−1^ min^−1^ and 5.39 mL kg^−1^ min^−1^ lower than Chinese Tibetan adolescents and Chinese Han adolescents at sea level (*p* < 0.05). The VO_2max_ scores of Chinese Tibetan adolescents are lower than Chinese Han adolescents at sea level at the age of 12–16 years old but higher than those at the age of 17 and 18 years old.

Table 3 shows the VO_2max_ scores of girls aged 12–18 for the three types of adolescents in China. Similar to boys, one-way ANOVA shows that there are significant differences in the VO_2max_ scores for the three types of adolescents at every age group except 16 years old (*p* < 0.01). For each type of adolescent, there are significant differences in VO_2max_ scores among different ages (*p* < 0.01). Overall, the VO_2max_ scores of Chinese Tibetan adolescents and Chinese Han adolescents at sea level were higher than Chinese Han adolescents born and raised at high altitudes, and the differences get bigger in 16–18 years old groups.

**Figure 1:**
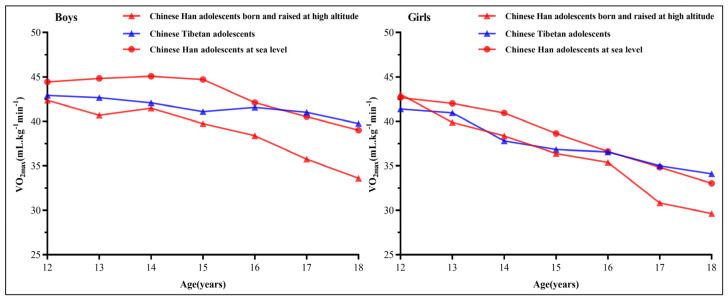
The changes of VO_2max_ (mL kg^−1^ min^−1^) scores with ages for the three types of adolescents aged 12–18 years old.

Figure 1 shows the changes in VO_2max_ scores with ages for Chinese Han adolescents born and raised at high altitudes, Chinese Tibetan adolescents, and Chinese Han adolescents at sea level. It should be noted that, for both boys and girls, the VO_2max_ scores of Chinese Tibetan adolescents exceeded Chinese Han adolescents at sea level after the age of 16 years old.

**Figure 2:**
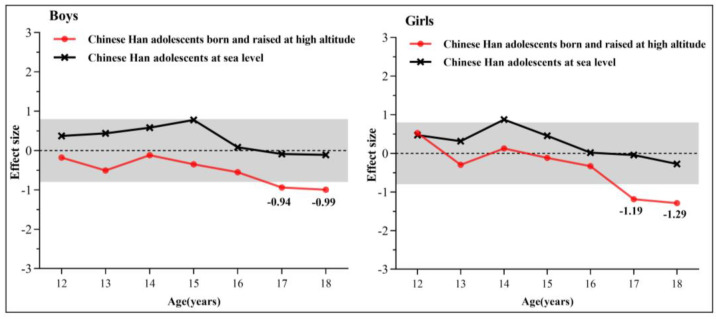
The effect size of VO_2max_ for Chinese Han adolescents born and raised at high altitudes and Chinese Han adolescents at sea level. Note: Effect sizes were obtained by taking the VO_2max_ means of Chinese Tibetan adolescents as references.

Figure 2 shows the effect size of VO_2max_ for Chinese Han adolescents born and raised at high altitudes and Chinese Han adolescents at sea level. In terms of boys, the effect size of the VO_2max_ score for Chinese Han at sea level between 12–18 years old is less than 0.8. For Chinese Han adolescents born and raised at high altitudes, the effect sizes are less than 0.8 at 12–16 years old groups but reached −0.94 and −0.99 at 17–18 years old group, which means that the VO_2max_ of Chinese Han adolescents born and raised at high altitudes is significantly lower than that of Chinese Tibetan adolescents. Similar patterns were found for girls, with effect sizes of Chinese Han adolescents born and raised at high altitudes at −1.19 and −1.29, respectively.

Table 4 shows the compared with Chinese Han adolescents at sea level, the VO_2max_ of Chinese Han adolescents born and raised at high altitude and Chinese Tibetan adolescents were negatively correlated with them (*p* < 0.01), and there was a dose-effect relationship, that is, the higher the altitude, the lower the VO_2max_.

## 4. Discussion

The present study compared the CRF of Chinese Tibetan adolescents with their Han counterparts born and raised at high altitudes and Chinese Han adolescents at sea level using the internationally recognized CFR evaluation method—20 m SRT. The result suggested that the VO_2max_ scores of Chinese Tibetan adolescents and Chinese Han adolescents at sea level were higher than Chinese Han adolescents born and raised at high altitudes. For both boys and girls, the VO_2max_ scores of Chinese Tibetan adolescents exceeded Chinese Han adolescents at sea level after the age of 16 years.

The 20 m SRT laps increased with ages for Chinese Han adolescents born and raised in Tibet of China, Chinese Tibetan adolescents, and Chinese Han adolescents at sea level aged 12–18 years old, regardless of sex. This result was consistent with our previous research, and an international study involved 1,142,026 adolescents across 50 counties around the world [33,34]. It is noteworthy that the 20 m SRT laps of Chinese Han adolescents born and raised at high altitudes decreased after the age of 16 years, especially for girls. The reasons for this result may be the decrease in physical activity level caused by high school academic pressure, or the low oxygen level in the body caused by a high-altitude hypoxia environment, etc. Overall, this result reminded us that Chinese Han adolescents born and raised at high altitudes aged 16–18 years old should be paid more attention, and targeted interventions should also be provided.

Our results also showed that the VO_2max_ level of Chinese Han adolescents born and raised at high altitudes was lower than Chinese Han adolescents at sea level and Chinese Tibetan adolescents, especially in the 15–18 age group for both boys and girls. The low VO_2max_ level of Chinese Han adolescents born and raised at high altitudes may be due to the influence of the hypoxic environment of Tibet of China. However, Chinese Han adolescents born and raised at high altitudes do not have an adaptation mechanism to altitude hypoxia-like Chinese Tibetan adolescents. A similar result was found in the hematological index: Garruto et al. [16] confirmed in their study that the hemoglobin concentration and hematocrit of Chinese Tibetan children and Chinese Han children born and raised at 3800 m and 4300 m were higher than the standard values in low-altitude areas, and there was almost no difference in hematological response patterns between Chinese Tibetan children and Chinese Han children born and raised at high altitude before the age of 13 years old. However, Chinese Han adolescents and adults have higher hemoglobin concentration and hematocrit than Chinese Tibetans adolescents and adults, regardless of sex. They concluded that the adolescent period might involve a divergence in the responses to hypoxia.

It is worth noting that our study also found that the VO_2max_ level of the Chinese Tibetan adolescents at the age of 12–15 was lower than their Chinese Han counterparts at sea level. While after the age of 15, the VO_2max_ level of the Tibetan adolescents gradually exceeded the Chinese Han at sea level concluded that Chinese Tibetans formed hypoxia adaptation compensatory mechanism and different body shape structures such as more deep and wider thorax compared with long-term living at high altitude [35]. Another study focused on the chest bone of Atacama Plateau residents found that the length and width of the sternum and clavicle were both higher than those of the inhabitants of lower elevations, which were shaped by plateau hypoxia environment [36]. In addition, there are differences in oxygen supply in the brains between Chinese Tibetan adolescents and Chinese Han adolescents in high-altitude areas. Compared with the Chinese Han adolescents in high-altitude areas, the brain tissue oxygenation of Chinese Tibetan adolescents is higher during extreme exercise in normoxia, while the muscle tissue oxygenation is lower during exercise in hypoxia, indicating that Chinese Tibetans privileged oxygenation of the brain at the expense of that of the muscle [37]. Finally, higher 20 m SRT test scores require larger body muscle strength, especially the muscle strength of lower limbs, which would directly affect the score of 20 m SRT test scores. A recent study showed that the performance of the standing long jump for Chinese Tibetan adolescents increased faster than the Chinese average level after the age of 15, which may result in the better CFR performance of Chinese Tibetan adolescents than Chinese Han adolescents at sea level [34].

It should be noted that Chinese Han born and raised at high altitudes have the lowest level of CRF. It was also reported that there were differences in lung function (including adjusted forced vital capacity, forced expiratory volume in one second, and forced expiratory flow at 50% of FVC) between Tibetans and Han Chinese who live at the same altitude [38]. The low effect size (<−0.8) of Chinese Han born and raised at high altitude at 17–18 years old groups represent that more attention should be paid to the Chinese Han born and raised at high altitude. Positive intervention measures should be taken to curb the decrease in CFR levels of Han adolescents in high-altitude areas to ensure the healthy development of their bodies.

The main strength of the present study is that we used the internationally recognized 20 m SRT test to evaluate the CFR of Chinese Han born and raised at high altitudes, Chinese Tibetan adolescents, and Chinese Han adolescents at sea level, which can provide data for future international comparations. One important limitation of the present study is its cross-sectional design, and longitudinal or cohort studies are needed to further verify our findings. Another limitation lies in the lack of information on children’s and adolescents’ lifestyles, such as physical activity, sleep, diet, and so on. Further studies and lifestyle surveys should be conducted to examine the influence of lifestyle factors on CRF in Chinese Tibetan adolescents.

## 5. Conclusions

The present study compared the CRF of Chinese Tibetan adolescents with their Han counterparts born and raised at high altitudes and Chinese Han adolescents at sea level using the internationally recognized 20 m SRT test to evaluate the CFR. The result suggested that the CFR of Chinese Tibetan adolescents and Chinese Han adolescents at sea level were higher than Chinese Han adolescents born and raised at high altitudes. For both boys and girls, the CFR of Chinese Tibetan adolescents exceeded Chinese Han adolescents at sea level after the age of 16. The results of the study suggest that we should focus on the changes in CRF in adolescents at different altitudes and should adopt different CRF interventions for adolescents at different altitudes.

## Figures and Tables

**Table 1:** 20 m SRT (number of laps) by age and sex for the three types of adolescents aged 12–18 years old(M ± SD).

Gender	Age (Years)	Sample Size	Chinese Han Adolescents Born and Raised at High Altitude	Sample Size	Chinese Tibetan Adolescents	Sample Size	Chinese Han Adolescents at Sea Level
Boys	12	62	26 ± 8	77	29 ± 13	79	34 ± 15
	13	63	27 ± 10	69	34 ± 16	66	42 ± 20
	14	64	35 ± 16	50	38 ± 18	88	48 ± 18
	15	58	35 ± 13	51	40 ± 14	75	53 ± 19
	16	76	36 ± 18	67	48 ± 21	78	49 ± 22
	17	61	34 ± 16	60	50 ± 20	95	49 ± 19
	18	68	31 ± 11	65	52 ± 25	75	48 ± 20
Girls	12	67	30 ± 13	52	23 ± 7	67	27 ± 10
	13	64	24 ± 14	58	27 ± 9	51	32 ± 12
	14	60	25 ± 16	56	24 ± 11	55	33 ± 10
	15	62	24 ± 10	69	26 ± 13	49	31 ± 11
	16	64	26 ± 10	63	30 ± 10	90	30 ± 10
	17	62	19 ± 7	71	30 ± 13	76	30 ± 9
	18	67	20 ± 8	56	33 ± 12	42	30 ± 12

Note: M, mean; SD, standard deviation.

**Table 2:** The VO_2max_ (mL kg^−1^ min^−1^) scores by age and sex for the three types of adolescents aged 12–18 years old (boys). (M ± SD).

Age		Sample Size	Chinese Han Adolescents Born and Raised at High Altitude	Sample Size	Chinese Tibetan Adolescents	Sample Size	Chinese Han Adolescents at Sea Level	*F*	*p*
12		62	42.37 ± 2.38	77	42.93 ± 3.71	79	44.44 ± 4.37 bc	6.213	0.002
13		63	40.69 ± 2.96	69	42.67 ± 4.63 a	66	44.84 ± 5.27 bc	14.205	0.000
14		64	41.50 ± 4.65	50	42.08 ± 5.29	88	45.07 ± 4.98 bc	11.310	0.000
15		58	39.73 ± 4.05	51	41.09 ± 3.77	75	44.71 ± 5.38 bc	21.204	0.000
16		76	38.37 ± 5.25	67	41.58 ± 6.32 a	78	42.11 ± 6.10 b	8.904	0.000
17		61	35.74 ± 5.03	60	41.03 ± 6.17 a	95	40.52 ± 5.53 b	17.542	0.000
18		68	33.60 ± 3.87	65	39.75 ± 7.83 a	75	38.99 ± 5.61 b	21.572	0.000
	*F*		37.749		2.586		16.504		
	*p*		0.000		0.018		0.000		

Note: M, mean; SD, standard deviation. LSD analysis, a: comparison between Chinese Han adolescents born and raised at high altitude and Chinese Tibetan adolescents; b: comparison between Chinese Han adolescents born and raised at high altitude and Chinese Han adolescents at sea level; c: comparison between Chinese Han adolescents at sea level and Chinese Tibetan adolescents; *p <* 0.05.

**Table 3:** The VO_2max_ (mL kg^−1^ min^−1^) scores by age and sex for the three types of adolescents aged 12–18 years old (girls). (M ± SD).

Age		Sample Size	Chinese Han Adolescents Born and Raised at High Altitude	Sample Size	Chinese Tibetan Adolescents	Sample Size	Chinese Han Adolescents at Sea Level	*F*	*p*
12		67	43.05 ± 3.89	52	41.40 ± 2.21 a	67	42.67 ± 3.14 c	4.114	0.018
13		64	39.88 ± 4.22	58	40.97 ± 3.06	51	42.02 ± 3.58 b	4.842	0.009
14		60	38.36 ± 4.81	56	37.80 ± 3.80	55	40.96 ± 3.39 bc	9.523	0.000
15		62	36.38 ± 3.16	69	36.84 ± 4.47	49	38.63 ± 3.32 bc	5.321	0.006
16		64	35.39 ± 3.15	63	36.56 ± 3.82	90	36.61 ± 3.09 b	2.925	0.056
17		62	30.81 ± 2.53	71	35.00 ± 4.29 a	76	34.84 ± 3.26 b	30.586	0.000
18		67	29.63 ± 3.02	56	34.09 ± 3.84 a	42	33.02 ± 3.95 b	26.156	0.000
	*F*		115.510		31.254		70.322		
	*p*		0.000		0.000		0.000		

Note: M, mean; SD, standard deviation. LSD analysis, a: comparison between Chinese Han adolescents born and raised at high altitude and Chinese Tibetan adolescents; b: comparison between Chinese Han adolescents born and raised at high altitude and Chinese Han adolescents at sea level; c: comparison between Chinese Han adolescents at sea level and Chinese Tibetan adolescents; *p* < 0.05.

**Table 4:** Linear regression analysis of VO_2max_ and altitude of adolescents aged 12–18 years.

Model	Independent Variable	*B*	Standard Error	*β*	*t*-Value	*p*-Value	*B* (95.0% *CI*)	
I	Constant Term	64.863	0.743		87.271	<0.01	63.406	66.320
	Gender	−3.905	0.183	−0.336	−21.303	<0.01	−4.265	−3.546
	Age	−1.314	0.046	−0.452	−28.695	<0.01	−1.403	−1.224
II	Constant Term	66.125	0.723		91.479	<0.01	64.707	67.542
	Gender	−3.781	0.177	−0.325	−21.412	<0.01	−4.127	−3.435
	Age	−1.316	0.044	−0.453	−29.886	<0.01	−1.402	−1.229
	Chinese Han adolescents born and raised at high altitude	−3.178	0.213	−0.257	−14.913	<0.01	−3.596	−2.760
	Chinese Tibetan adolescents	−1.193	0.215	−0.095	−5.542	<0.01	−1.615	−0.771

Note: Model I controlled age and gender, and Model II added altitude factors for analysis on the basis of Model I. After adjustment of model I, *R*^2^ = 0.318, *p* < 0.01; after adjustment of model II, *R*^2^ = 0.370, *p* < 0.01.

## Data Availability

To protect the privacy of participants, the questionnaire data will not be disclosed to the public. If necessary, you can contact the corresponding author.
